# Ground Water Awareness Week — March 10–16, 2013

**Published:** 2013-03-08

CDC is collaborating with the National Ground Water Association to highlight National Ground Water Awareness Week, March 10–16, 2013. Water is essential for life. However, many persons are not aware that much of the water they use flows from below ground to the surface. The National Ground Water Association uses this week to stress ground water’s importance to the health and well-being of humans and the environment (1).

The majority of public water systems in the United States use ground water as their primary source, providing drinking water to nearly 90 million persons (2). An additional 15 million U.S. homes use private wells, which also rely on ground water (3).

Usually, ground water in the United States is safe to use. However, ground water sources can be contaminated naturally or as a result of imperfect agricultural, manufacturing, or sanitation practices (4). The presence of contaminants, such as pesticides, factory waste, and sewage, can lead to acute and chronic illness (5).

The U.S. Environmental Protection Agency has worked with individual states to develop new regulations to provide increased protection against microbial pathogens in public water systems that use ground water sources (6). Private ground water wells serving fewer than 25 persons might not be regulated but nonetheless must be properly maintained by well owners to ensure that the water remains free from harmful chemicals and pathogens.* Resources are available from state and local health departments to help homeowners protect their ground water.†

## Footnotes

* Additional information available at http://www.cdc.gov/healthywater/drinking/private/wells/index.html.

† Additional information available at http://www.apha.org/about/public+health+links/linksstateandlocalhealthdepartments.htm.

## References

[1] National Ground Water Association (2012). National Ground Water Awareness Week: March 10–16, 2013.

[2] US Environmental Protection Agency (2012). Fiscal year 2010 drinking water and ground water statistics.

[3] US Census Bureau (2011). Current housing reports, series H150/09, American housing survey for the United States: 2009.

[4] CDC (2011). Surveillance for waterborne disease outbreaks associated with drinking water—United States, 2007–2008. MMWR.

[5] US Environmental Protection Agency (2012). Drinking water contaminants.

[6] US Environmental Protection Agency (2012). Ground water rule (GWR).

